# Endophthalmitis Outbreak Associated with Repackaged Bevacizumab

**DOI:** 10.3201/eid2101.141040

**Published:** 2015-01

**Authors:** Laura S. Edison, Hope O. Dishman, Melissa J. Tobin-D’Angelo, C. Richard Allen, Alice Y. Guh, Cherie L. Drenzek

**Affiliations:** Centers for Disease Control and Prevention Atlanta, Georgia, USA (L.S. Edison, A.Y. Guh);; Georgia Department of Public Health, Atlanta (L.S. Edison, H.O. Dishman, M.J. Tobin-D’Angelo, A.Y. Guh, C.L. Drenzek);; Georgia Drug and Narcotics Agency, Atlanta (C.R. Allen)

**Keywords:** Gram-positive, outbreak, drug compounding, drug contamination, endophthalmitis, eye infections, humans, intravitreal injections, vascular endothelial growth factor A, bacteria, Georgia, Indiana, United States, eye, ocular

**To the Editor:** An outbreak of endophthalmitis associated with repackaged bevacizumab occurred during February–March 2013 in Georgia and Indiana, USA. Bevacizumab (Avastin; Genentech, Inc., South San Francisco, CA, USA) is a vascular endothelial growth factor inhibitor that is approved by the US Food and Drug Administration as an antineoplastic agent but is commonly used off-label to treat retinal disorders, including age-related macular degeneration (*1*,*2*). Bevacizumab is manufactured in single-use, preservative-free, 4-mL vials; compounding pharmacies repackage bevacizumab into syringes for intraocular administration at smaller doses (e.g., 1.25 mg bevacizumab in 0.05-mL injection). Repackaged bevacizumab has been linked to endophthalmitis outbreaks worldwide in which compounding procedure deficiencies have led to microbial contamination and subsequent endophthalmitis (*3*–*7*). Endophthalmitis often results in vision loss, particularly if the infection is not identified early and treated aggressively (*4*–*6*). 

During March 6–8, 2013, four patients with age-related macular degeneration received a diagnosis of acute endophthalmitis after receiving intravitreal bevacizumab injections on March 4, 2013, at a retinal specialty clinic (clinic A) in Georgia. All 4 patients were injected with bevacizumab from the same lot (lot Z), which was repackaged at a Georgia compounding pharmacy (pharmacy A) on February 13, 2013. The Georgia Department of Public Health (DPH) and the Georgia Drug and Narcotics Agency (GDNA) were notified of the outbreak by clinic A, and the outbreak was investigated to determine the extent and source of infections and to prevent additional cases.

Cases were defined as acute endophthalmitis occurring among patients <14 days after they received intraocular injection with bevacizumab that had been repackaged at pharmacy A after December 18, 2012. Pharmacy A records indicated that bevacizumab had been distributed to 11 clinics in 4 states; state public health authorities were notified, and facilities that received the medication were contacted and asked to report cases to DPH. Clinic A identified 60 additional patients who received bevacizumab from lot Z and monitored them for signs of infection. One additional patient with endophthalmitis was identified in Indiana; this patient was injected on February 22 with bevacizumab that had been repackaged at pharmacy A on February 13, 2013.

DPH epidemiologists evaluated clinic A infection prevention practices, including use of face masks and sterile techniques during injection procedures; examined possible sources of contamination; and reviewed case-patient medical records. DPH and GDNA evaluated pharmacy A, including its equipment and sterile compounding procedures. Vitreal fluid samples were collected from all case-patients and were cultured on chocolate agar medium.

The median age of the 5 case-patients was 80 years (range 59–89 years); 3 case-patients were men. Postprocedural signs and symptoms included pain, vision loss, retinal hemorrhage, hypopyon, and vitreous haze. Patients who received a diagnosis of endophthalmitis were treated with pars plana vitrectomy or vitreal tap; intraocular injection of vancomycin with gentamicin, ceftazidime, or ceftazidime and amphotericin; and oral moxafloxacin. All patients regained vision in the affected eye.

Evaluation of clinic A found appropriate mask and glove use and no deficiencies in bevacizumab injection technique or medication storage and handling. Investigation of pharmacy A revealed multiple areas in which practices did not conform to United States Pharmacopeial Convention Chapter 797 standards for compounding sterile preparations or to recommended best practices for repackaging bevacizumab; these deficiencies might have contributed to bevacizumab contamination (*6*,*8*). GDNA suspended pharmacy A from performing sterile compounding until compliance with these standards.

Culture of vitreous fluid samples from all patients in Georgia grew *Granulicatella adiacens*, a gram-positive bacterium that is part of the oral flora but a rare human pathogen not previously reported to cause endophthalmitis (*9*). The Indiana patient was infected with *Abiotrophia* (not further speciated); *Granulicatella* and *Abiotrophia* are similar bacterial genera that are difficult to distinguish by morphologic features (*10*).

Contamination introduced during repackaging at pharmacy A was the likely source of this outbreak. This conclusion was supported by evidence of common or similar organisms among 5 patients from 2 states after injection with bevacizumab repackaged on the same date at pharmacy A, combined with documented deficiencies in pharmacy A’s sterile compounding processes.

Acute postinjection endophthalmitis can occur when intraocular bevacizumab is used because of risks associated with repackaging contents from single-use, preservative-free vials. Ensuring adherence to standards for sterile compounding is critical for preventing contamination and providing patients with a safe source of intraocular bevacizumab (*6*). If this drug were available from the manufacturer in appropriately sized, prefilled syringes or containers, risks associated with repackaging and mishandling might be eliminated (*2*,*6*). Ophthalmology clinics that rely on repackaged bevacizumab for intraocular use should be vigilant in selecting pharmacies to perform this service. Resources are available to assist facilities in assessing the quality of outsourced sterile compounding services (e.g., http://www.ashpfoundation.org/MainMenuCategories/PracticeTools/SterileProductsTool/SterileProductsAssessmentTool.aspx). In addition, ophthalmologists should adhere to aseptic techniques and safe injection practices (http://www.cdc.gov/injectionsafety/ip07_standardprecaution.html) when preparing or administering intraocular injections. A single case of endophthalmitis may signal a more widespread problem; prompt reporting to public health authorities, investigation, clinician engagement, and product recalls can be critical for limiting patient harm.
